# Targeting SOST using a small-molecule compound retards breast cancer bone metastasis

**DOI:** 10.1186/s12943-022-01697-4

**Published:** 2022-12-29

**Authors:** Lisha Sun, Yixiao Zhang, Guanglei Chen, Yaoting Ji, Qingtian Ma, Xinbo Qiao, Sijin Wu, Lin Zhou, Jiawen Bu, Xudong Zhu, Xiaoying Zhang, Xiaofan Jiang, Chao Liu, Xinnan Li, Yang Liu, Yongliang Yang, Caigang Liu

**Affiliations:** 1 Department of Oncology, Innovative Cancer Drug Research and Engineering Center of Liaoning Province, Cancer Stem Cell and Translation Medicine Lab, Shengjing Hospital of China Medical University, Shenyang, China; 2 Department of Urology Surgery, Shengjing Hospital of China Medical University, Shenyang, China; 3 The State Key Laboratory Breeding Base of Basic Science of Stomatology (Hubei-MOST) and Key Laboratory for Oral Biomedicine of Ministry of Education, School and Hospital of Stomatology, Wuhan University, Wuhan, China; 4 Dalian Institute of Chemical Physics, Chinese Academy of Science, Dalian, China; 5 Key Laboratory of Structure-Based Drug Design & Discovery of Ministry of Education, Shenyang Pharmaceutical University, Shenyang, China; 6 School of Bioengineering, Dalian University of Technology, Dalian, China

**Keywords:** SOST, Bone metastasis, Breast cancer, Small-molecule compound

## Abstract

**Background:**

Breast cancer metastasis to the bone can be exacerbated by osteoporosis, is associated with poor long-term survival, and has limited therapeutic options. Sclerostin (SOST) is an endogenous inhibitor of bone formation, and an attractive target for treatment of osteoporosis. However, it is unclear whether SOST can be used as a therapeutic target for bone metastases of breast cancer, and whether small molecule compounds that target SOST in breast cancer cells can inhibit breast cancer bone metastasis.

**Methods:**

SOST expression in 442 breast cancer tissues was characterized by immunohistochemistry and statistically analyzed for the association with breast cancer bone metastases. Bone metastatic breast cancer SCP2 cells were induced for SOST silencing or overexpression and their bone metastatic behaviors were tested in vitro and in vivo. To identify potential therapeutics, we screened inhibitors of the interaction of SOST with STAT3 from a small chemical molecule library and tested the inhibitory effects of one inhibitor on breast cancer growth and bone metastasis in vitro and in vivo.

**Results:**

We found that up-regulated SOST expression was associated with breast cancer bone metastases and worse survival of breast cancer patients. SOST silencing significantly reduced the bone metastatic capacity of SCP2 cells. SOST interacted with STAT3 to enhance the TGF-β/KRAS signaling, increasing both tumor growth and bone metastasis. Treatment with one lead candidate, S6, significantly inhibited the growth of breast-cancer organoids and bone metastasis in mice.

**Conclusions:**

Our findings highlight a new class of potential therapeutics for treatment of bone metastasis in breast cancer.

**Supplementary Information:**

The online version contains supplementary material available at 10.1186/s12943-022-01697-4.

## Background

Cancer bone metastasis occurs in more than 1.5 million patients per year worldwide [1]. Patients with cancer bone metastasis usually suffer from severe skeletal-related events (SREs), including bone fractures, severe pain, and disability, which seriously decreases the quality of life [2]. It has been well documented that osteoporosis is a risk factor for cancer bone metastases [3] and anti-osteoporosis drugs have been used as the conventional treatment of patients with cancer bone metastasis.

Bone targeted agents (BTAs), such as bisphosphonates, can reduce osteolysis and improve bone microenvironments to inhibit the progression of cancer bone metastasis. However, bisphosphonates failed to prevent bone metastases in early breast cancer in the SUCCESS, a phase III clinical trial [4]. Although bisphosphonates have been reported to inhibit the proliferation of breast cancer cells [5, 6], there is no evidence to demonstrate that bisphosphonates can directly eliminate cancer cells in focal bone lesions. In addition, a number of osteosecretory proteins are important for the development of osteoporosis and therapeutic humanized antibodies against those proteins have been developed with the aim of inhibiting bone resorption and metastasis. Denosumab is an antibody against the receptor activator of nuclear factor kappa-B ligand (RANKL). Treatment with denosumab can delay the onset of SRE and subsequent (multiple) progression in patients with cancer bone metastasis [7]. However, there remains no evidence that denosumab directly eliminates cancer cells in focal bone lesions.

Sclerostin (SOST) is a glycoprotein secreted by mature osteocytes in the bone matrix [8]. Functionally, SOST acts as an inhibitor of bone formation, making it a therapeutic target for the treatment of osteoporosis [9–12]. Furthermore, SOST acts as oncogenic factor to induce breast cancer bone metastasis as well as osteolysis and inhibition of SOST alleviates the breast cancer-induced bone metastasis and muscle weakness [13, 14]. Romosozumab is a humanized antibody against SOST and has been approved by the US FDA for the treatment of osteoporosis [15]. Functionally, romosozumab can bind to SOST in the stroma to promote osteogenesis [16]. While it has excellent efficacy in the treatment of postmenopausal osteoporosis, it was associated with high cardiovascular risk in clinical trials [17, 18]. Hence, discovery of new inhibitors of SOST, particularly for small chemical molecules, is urgently needed for the treatment of cancer bone metastasis and osteoporosis safely.

In this study, we found that up-regulated SOST expression was associated with worse prognosis in breast cancer patients. Mechanistically, SOST promoted the proliferation and bone metastasis of breast cancer cells by activating downstream signaling pathways. Using computational screening, we identified a candidate therapeutic compound that disrupted the interaction of SOST with STAT3 to inhibit STAT3 phosphorylation and reduce breast cancer bone metastasis.

## Methods

### Patients

A cohort of 422 patients with breast cancer and 69 pairs of patients with primary breast foci with metastatic lesions were recruited at the Shengjing Hospital of China Medical University from April 2011 to July 2013. Patients with breast cancer were diagnosed based on radiological and pathological examinations. Individual breast cancer patients were excluded if they had incomplete clinical data, received neoadjuvant chemotherapy or radiotherapy, another type of malignant tumor, severe organ dysfunction, or bilateral breast cancer. Surgical tumor tissues were collected, fixed in 10% formalin, and paraffin-embedded for histological examination and immunohistochemistry (IHC). Written informed consent was obtained from all patients. The experimental protocol was approved by the Institutional Research Ethics Committee of Shengjing Hospital of China Medical University (Project ID 2018PS304K, approved on 03/05/2018).

### Histology

The paraffin-embedded tissue Sects. (4 μm) were dewaxed and rehydrated, followed by hematoxylin and eosin (H&E) staining. The sections were photographed under a light microscope (Olympus, Tokyo, Japan) and examined independently by two pathologists in a blinded manner.

### Immunohistochemistry

Tissue Sects. (4 μm) were dewaxed, rehydrated, and treated with 3% H_2_O_2_ in methanol, followed by antigen retrieval in citrate buffer (pH 6.0) in a microwave for 10 min. After being blocked with 5% bovine serum albumin (BSA) in Tris-buffered saline with Tween® 20 (TBST), the sections were probed with mouse anti-SOST (ab63097, Abcam, Cambridge, MA, USA) at 25 ℃ for 2 h. The bound antibodies were detected using horseradish peroxidase (HRP)-conjugated goat anti-mouse immunoglobulin IgG at room temperature for 30 min and visualized using 3,3′-diaminobenzidine, followed by counterstaining with hematoxylin. The sections were mounted and photographed under a light microscope. The intensities and frequencies of positively stained cells were evaluated using IHC Profiler software [19].

### Cell culture

Human breast cancer MDA-MB-231, MCF-7 cells, and mouse osteogenic precursor cells (MC3T3-E1) were obtained from the American Type Culture Collection (Manassas, VA, USA). SCP2 cells were a gift from Professor Joan Massague (Cell Biology Program and Howard Hughes Medical Institute, Memorial Sloan-Kettering Cancer Center, NY, USA). MDA-MB-231 cells were cultured in Leibovitz’s L15 medium (Thermo Fisher, Carlsbad, CA, USA), and MCF-7 cells were cultured in Dulbecco’s modified Eagle’s medium (DMEM) containing 10% of fetal bovine serum (FBS, Biological Industries, Cromwell, CT, USA). MC3T3-E1 cells were cultured in α-MEM plus 10% of FBS and 1% P/S. All cells were incubated at 37 °C in a humidified atmosphere with 5% CO_2_.

### Transduction

SCP2 cells (10^7^/well) were transduced with lentivirus at a multiplicity of infection of 10 for the expression of control shRNA or *SOST*-specific shRNA (with green fluorescent protein (GFP), Sangon Biotech, Shanghai, China) in the presence of 5 µg/mL puromycin (A1113803, Thermo Fisher) for 7 days to establish stable SCP2/NC and *SOST*-knockdown SCP2/KD cells. The efficacy of *SOST* silencing was determined by Western blotting. The shRNA target sequences were: *SOST* KD1, 5’-GCAGTGAAAGATGTAGCCAAA-3’ and *SOST* KD2, 5’-GCCTCAGATAATCTGGTGAAA-3’. We retrieved the SOST gene sequence from GenBank and designed the primers: *SOST*-F (*Eco*RI): AGGGAGACCCAAGCTGGCTAGTTGaattcGCCACCATGCAGCTCCCACT, *SOST*-R (*Bam*HI): GTCACTTAAGCTTGGTACCGAggatccGTAGGCGTTCTCCAGCTCGGC. In addition, the cDNA fragment of *SOST* was cloned into pcDNA3.1-CMV-MCS-3flag-EF1-ZsGreen-T2A-Puro vector and sequenced. MDA-MB-231 cells were transfected with control plasmid or the SOST-expressing plasmid and selected by treatment of cells with G418 to establish stable SOST over-expressing MDA-MB-231 (MDA-MB-231/OE) cells or control MDA-MB-231/NC cells. The levels of SOST expression were quantified by Western blot.

### Quantitative real-time reverse transcription-polymerase chain reaction (qRT-PCR)

Total RNA was extracted from SCP2/KD, SPC2/NC, MDA-MB-231/OE or MDA-MB-231/NC cells using TRIzol reagent (Thermo Fisher). The RNA samples were reverse transcribed into cDNA. The relative levels of gene mRNA transcripts to the control GAPDH were quantified by qRT-PCR using PrimeScript RT Master Mix (RR047A, Takara, Kyoto, Japan) and TB Green Premix Ex Taq II (RR820A, Takara), according to the manufacturer’s instructions. Data were analyzed by 2^−ΔΔCt^.

### Western blotting

MDA-MB-231, SCP2, MCF-10A, SCP2/NC, and SCP2/KD cells were lysed in radioimmunoprecipitation assay (RIPA) buffer and centrifuged (1000 × *g* at 4 °C for 20 min). After determining protein concentrations using a bicinchoninic acid assay kit (Thermo Fisher), the cell lysates (40 µg/lane) were separated by sodium dodecyl sulfate polyacrylamide gel electrophoresis (SDS-PAGE) on 12% gels and transferred to polyvinylidene fluoride membranes (MilliporeSigma, Burlington, MA, USA). The membranes were blocked with 5% BSA in TBST and incubated with primary antibodies at 4 °C overnight. The primary antibodies included anti-SOST (ABIN6997488, antibodies-online GmbH, Germany), anti-TGF-β (ER31210, HuanBio, Hangzhou, China), anti-SMAD3 (66,516–1-1 g, PTG, Rosemont, USA), anti-CXCR4 (60,042–1-1 g, PTG), anti-STAT3 (10,253–2-AP, PTG), anti-p-STAT3 (9138, Cell Signaling Technology), anti-KRAS (12,063–1-AP, PTG), and anti-β-actin (20,536–1-AP, PTG). The bound antibodies were detected using HRP-conjugated secondary antibodies (1:10,000; Jackson ImmunoResearch Laboratories, West Grove, PA, USA). The immune signals were visualized using enhanced chemiluminescence reagent (Thermo Fisher). The relative levels of individual target proteins to β-actin were determined by densitometric analysis using ImageJ software (US National Institutes of Health, Bethesda, MD, USA).

### Immunoprecipitation

SCP2 cells were harvested and lysed with cold RIPA lysis buffer containing protease inhibitors, followed by centrifuging. The cell lysates (50 µg/tube) were reacted with anti-SOST, anti-STAT3, or control isotype IgG (2 µg) with gentle agitation at 4 ºC overnight. In addition, the cells lysates (1 ml) were pre-treated with 200 μM S6 compound at room temperature for one hour and reacted with each type of antibodies. The generated immunocomplexes were precipitated with 20 µl of protein A/G plus-agarose beads (sc-2003, Santa Cruz, USA) at 4 ºC for 4 h. After being centrifuged, the palleted beads were washed with TBST and the bound proteins were eluted with 2 × SDS loading buffer. The eluted proteins were analyzed by SDS-PAGE and stained with anti-STAT3 or anti-SOST to detect the direct interaction of SOST with STAT3.

### Enzyme-linked immunosorbent assay (ELISA)

SCP2 cells were co-cultured with MC3T3-E1 cells (5 × 10^5^ cells/well) in 6-well plates and treated with DMSO or S6 compound for 24 h. The levels of CXCL12 in the supernatants of cultured cells were determined using an ELISA kit (KE10049, PTG) and 3,3',5,5'-tetramethylbenzidine, according to the manufacturer’s instructions. The experimental samples were tested in triplicate. The absorbance of each well was measured at 450 nm using a microplate reader (Biotek, USA). The CXCL12 concentrations were calculated using a standard curve established with recombinant CXCL12 protein provided.

### Chemotaxis assay

Breast cancer cells (3 × 10^4^/chamber) were suspended in 2% of FBS medium (200 µl) and cultured in the top chamber of 24-well transwell plates (3422, Corning, USA). The bottom chamber was cultured with MC3T3-E1 cells up to 80% confluence in 200 µl of medium containing 2% of FBS. The top and bottom chambers were co-cultured for 24 h. The cells on the upper membrane of the top chamber were removed using a cotton ball while the cells that migrated the bottom surface of the top chamber membrane were stained with 0.5% (v/m) crystal violet and counted in a blinded manner.

### Adhesion assay

To mimic the bone matrix, MC3T3-E1 cells were induced for osteogenic differentiation in MEM-α media containing 10% of FBS, 10 mM β-glycerophosphate (Solarbio), and 50 µg/ml of ascorbic acid (Solarbio) for 9 days. Subsequently, the cultured MC3T3-E1 cells were treated with 20 mM NH_4_OH (Sigma, USA) and 0.5% Triton X-100 (Solarbio) for 5 min to form the bone matrix layer. GFP expressing breast cancer cells (2 × 10^5^ cells/well) were added into each bone matrix layer and incubated for 15 min. After aspirating floating cells, the adhering cells were washed with PBS and counted under a fluorescent microscope.

### Cell viability and cytotoxicity assay

Viability and cytotoxicity were analyzed using a Cell Counting Kit-8 (CCK8) (Dojindo, Japan), according to the manufacturer’s instructions. SCP2/WT, SCP2/NC, SCP2/KD1, SCP2/KD2, MDA-MB-231 and MCF-7 cells were cultured in 96-well plates for 24, 48 or 72 h in the presence or absence of different concentrations of S6 compound or positive controls of 50 μM epirubicin (EADM) or 5 nM docetaxel (DTX). During the last 4-h culture, the cells were exposed to 10% CCK8 solution. The absorbance at 450 nm in individual wells was measured using a microplate reader (Biotek, USA).

### Biolayer interferometry (BLI)

The affinity of S6 binding to SOST was measured, as described previously [20] using an Octet K2 instrument (ForteBio). All assays were run at 30 ºC with continuous shaking of 1000 RPM using the assay buffer of 0.1% BSA, 0.01% Tween-20 and 1% DMSO in PBS. SOST (10,593-H07H, SinoBio, Beijing, China) at 0.15 mg/ml was dissolved in sterile water, biotinylated and immobilized onto the Octet SSA biosensors. S6 (C29H24N6O2S, molecular weight 520.62, Chemdiv, USA) was dissolved in PBS and adjusted to different concentrations for BLI. After each round of association and disassociation, the SOST-contained sensors were washed with the assay buffer for 10 min to remove nonspecifically bound molecules. Raw kinetic data were obtained. The k_on_ and k_off_ values were analyzed using the software provided and the *K*_d_ values were calculated, based on double reference subtraction.

### Murine xenograft tumor model

A mouse model of xenograft breast tumors was established, as previously described [21]. Female BALB/c nude mice were injected with 10^6^ SCP2/WT, SCP2/NC or SCP2/KD cells via the left ventricle (*n* = 8–10 per group) and their body weights were monitored for 42 days after inoculation. The bone metastatic colonization of breast tumor cells was monitored by X-rays and bioluminescence weekly, and the survival of tumor-bearing mice was checked daily.

For the drug treatment assay in vivo, individual mice receiving any type of SCP2 cells were randomized and treated intravenously with vehicle (5% of DMSO in PBS) or 10 mg/kg S6 twice per week for 42 days (*n* = 8–10 per group). Individual tumor cell-bearing mice were monitored longitudinally, and their body weights were measured every other day. The mice were anesthetized and euthanized when the mice lost 20–25% of their original body weights or developed symptoms of cachexia or wasting. The tumors in the bone and vertebrae were dissected and microCT photographed, followed by fixation, decalcification, embedding and H&E staining. The tumor tissue sections were stained with Tartrate-resistant acid phosphatase (TRAP) using a specific kit (387A, Sigma). Their heart, liver, spleen, lung, bone marrow and kidney tissues were collected, sectioned, and stained with H&E to observe potential S6 toxicity. The animal experiments were carried, per the Guide for the Care and Use of Laboratory Animals of National Institutes of Health Guide and the protocols were approved by the Institutional Research Ethics Committee of Shengjing Hospital of China Medical University (Project ID 2020PS318K, approved on 04/01/2020).

### Molecular modeling and docking

The intact structure of the major domains (A135-L731) of STAT3 was established, based on the crystal structures (Protein Data Bank [PDB] code 6QHD) and Alphafold2 model of STAT3 (UniProt ID P40763) using Modeller 9.22 with 1,000 decoys. The structure of STAT3 was evaluated using DOPE, Molpdf score, DFIRE2, and Procheck, followed by 50-ns molecular dynamics (MD) relaxation.

The loop1 and loop3 of the SOST NMR structure (PDB code 2K8P) of two beta-sheet structures of SOST protein were selected with the relaxed STAT3 modeling structure, residues 75–81 and 132–138, respectively. Rigid-body docking for SOST with STAT3 was performed with Zdock program to obtain the potential protein–protein interaction poses, with some key residues preferred during the docking process. The complex with the best docking score was used for 100-ns MD optimization and relaxation.

All MD simulations were performed using Gromacs 2020.4 with Amber14 force field. The structure was solvated in a cubic TIP3P water box with 1 nm distance from the edge, which was neutralized by adding appropriate number of sodium and chloride ions. After two steps of energy minimization, this system was gradually heated to 300 ºK over 100 ps to perform the 2-ns NVT equilibration and 5-ns NPT equilibration. Finally, MD simulations at 300 ºK and 1 atm were carried out with the LINCS algorithm to restrain hydrogen positions at their equilibrium distances, which allowed the use of an integration time step of 2 fs. Both energies and coordinates were saved every 10 ps for postproduction analysis. All simulations were performed on a high-performance computer cluster running the Linux operating system. The MM-PBSA calculation was conducted using the gmx_MMPBSA tool [22].

### Virtual screening

The final frame of the modeling structure from the MD simulation was used for site prediction and virtual screening. FTSite [23] and FTMap [24] were used to detect the binding pocket of SOST. Our in-house docking program, FIPSDock [25], was used to screen a library against SOST. The geometric center of the SOST structure was chosen as the grid center. Each grid contained 200 × 200 × 160 grid points with 0.375 Å spacing to cover the whole SOST structure. To narrow down the virtual screening results effectively, we chose only the best docked structures with the lowest binding free energies for each possible binding mode to estimate the binding pose and important interactions. Notably, the S6 molecule stood out among the top 200 hits. The complexed structure of SOST with the best pose of S6 was built to conduct 500-ns MD simulation.

### Testing drug sensitivity in organoid cultures

Surgical breast tissues were obtained from a patient with breast cancer at Shengjing Hospital of China Medical University after the patient provided informed consent. The tissues were minced using a scalpel, cultured into a 50-mL C-tube containing 20 mL of AdDF +  +  + (Advanced DMEM/F12 with 1 × Glutamax, 10 mM HEPES, and 1% P/S) and 2 mg/mL collagenase at 37 °C for 2 h with general shaking. After digestion, the suspension was centrifuged at 400 g for 10 min; mechanically sheared by pipetting with 10, 5, and 1 mL pipette tips; and resuspended in 10 mL of AdDF +  +  + containing 2% of FBS each time to obtain organoids. The cell suspension was added to Matrigel at a 1:2 ratio; 100 µL of the solution was then placed at the center of each well of a 24-well culture plate; the gel was solidified at 37 °C for 30 min in 5% CO_2_. The medium was replaced every 3 days and organoids were passaged weekly. The growth of organoids was evaluated under an inverted microscope, and the organoids with fine growth conditions that had been more than 3 passages were used for subsequent experiments. The organoids were treated with, or without (NS), 2 μM S6, or 80 μM S6 for 48 h and the potential toxicity was evaluated under an inverted microscope (Nikon, Japan).

### Differentiation of bone marrow mesenchymal stem cells (BMSCs)

To induce osteoblast differentiation, the isolated mouse BMSCs (2 × 10^5^ cells/well) were treated with DMSO or S6 (8 µM) in a modified α-MEM containing 10% of FBS, 50 μg/ml L-ascorbic acid, 1 mM dexamethasone and 1 M β-glycerophosphate in 12-well plates. The media were changed every other day for 14 days. The cells were fixed with 4% paraformaldehyde, permeabilized with 0.1% of Triton-X 100, denatured again with 0.1% PBST, and stained with alkaline phosphatase (ALP) using a specific kit (Sigma-Aldrich, USA). Some cells were cultured for 21 days and stained with alizarin red staining (ARS). Subsequently, the culture plates were washed with distilled water and treated with 10% of cetylpyridinium chloride for 2 h with general shaking to fully dissolve the mineralized nodules. The relative absorbance value of S6 to the control (DMSO) solution (designated as 1) was evaluated and calculated.

### Transcriptome sequencing and promoter prediction

SCP2/NC or SCP2/KD1 cells were harvested, and their total RNA was extracted, followed by transcriptome sequencing on illumina platform (Biomarker Technologies, Beijing, China). The levels of original gene mRNA transcripts were analyzed using the FPKM method and DESeq2. The differentially expressed genes (DEGs) were defined when log2FC > 1 or <  − 1, and false detection rate (FDR) < 0.05. The potential pathways of DEGs were analyzed by KEGG pathway annotation. The STAT3 binding sites in the *TGF-β* and *KRAS* promoter regions were predicted using rVista 2.0 software in JASPAR database [26].

### Statistical analysis

Data are expressed as means ± standard error of mean (SEM). Differences among groups were analyzed by one-way analysis of variance (ANOVA) and post hoc Newman-Keuls test. Differences between groups were analyzed by Student’s *t*-test. DMFS in each group of patients was estimated using the Kaplan–Meier method and analyzed by the log-rank test. Risk factors associated with poor survival were identified using univariate and multivariate regression analyses with the Cox regression model. All statistical analyses were performed using SPSS 23.0 software (IBM Corps., Armonk, NY, USA). Statistical significance was set at a *P-value* of < 0.05.

## Results

### High SOST expression is associated with breast cancer bone metastasis

**Fig. 1:**
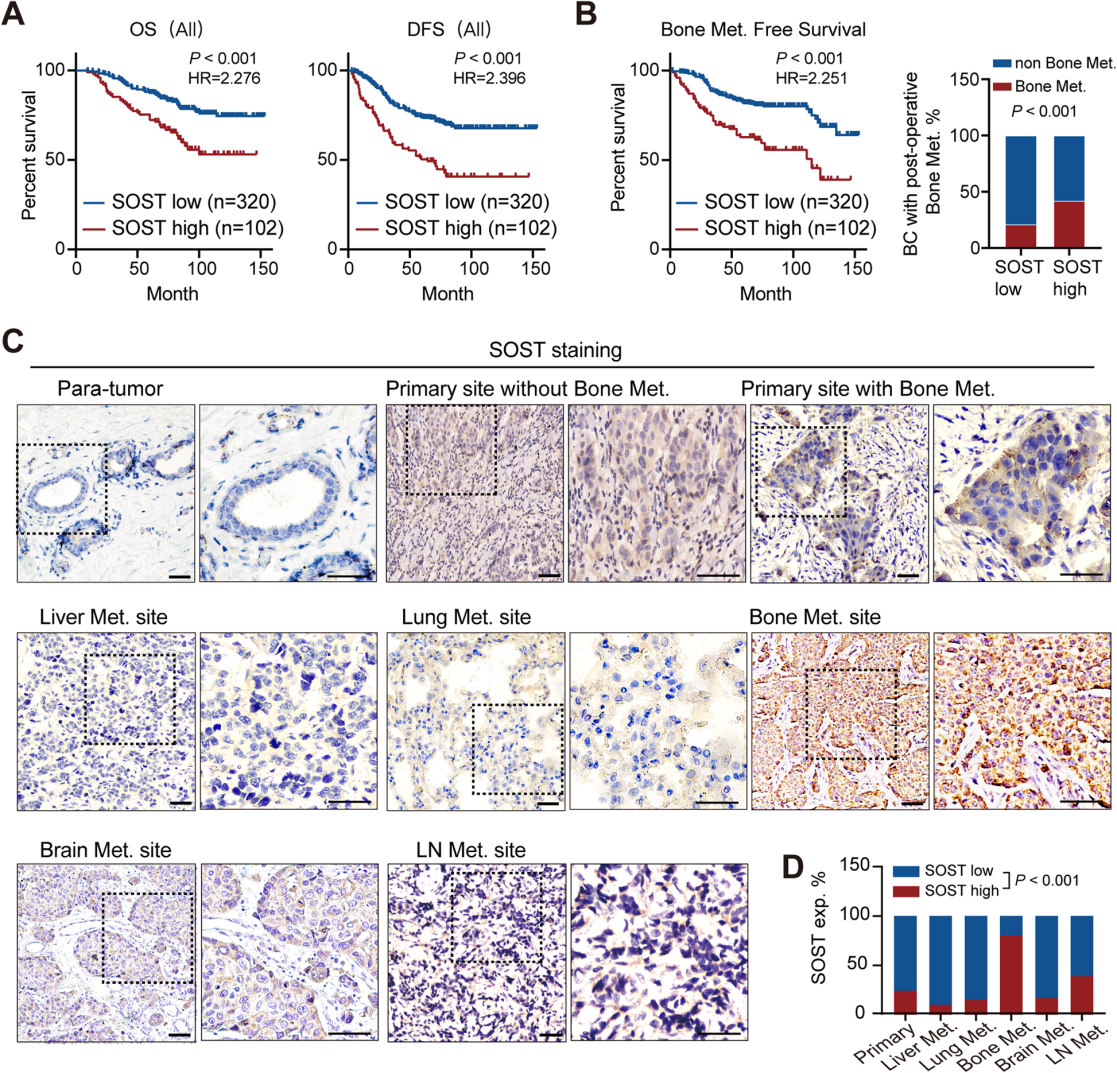
Up-regulated SOST expression is associated with bone metastasis and worse prognosis of breast cancer patients. **A** Overall survival (OS) and disease-free survival (DFS) of patients with high or low SOST expressing breast cancer (*P* < 0.001). Significance was determined by two-sided log-rank test. **B** Bone metastasis-free survival (BMFS) of patients with high or low SOST expressing breast cancer and the proportion of bone metastases within each group (*P* < 0.001). Significance was determined by two-sided log-rank test. **C** IHC micrographs showing SOST expression in normal breast tissue, primary breast tumors (*n* = 422), liver metastasis (*n* = 10), lung metastasis (*n* = 20), bone metastasis (*n* = 15), brain metastasis (*n* = 15), and lymph-node metastasis (*n* = 15); scale bar, 100 µm. **D** Statistical analysis of SOST expression corresponding to C

Identification of phenotypic characteristics of breast cancer bone metastasis is the most efficient approach to management of bone metastasis. First, we characterized SOST protein expression in primary foci of 422 breast cancer specimens by immunohistochemistry (IHC). High levels of SOST expression were detected in 24.2% (102/422) of the breast cancer tissues and significantly associated with worse overall and disease-free survival (*P* < 0.001, Fig. 1A). Stratification analysis of different molecular types of breast cancers revealed the same trend (Fig. S1A-B), as did the triple-negative breast cancer (TNBC) subgroup from The Cancer Genome Atlas (TCGA) (Fig. S1C).

Further analysis indicated that patients with high SOST expressing tumors tended to have a shorter bone metastasis-free survival; moreover, those patients were more likely to develop bone metastasis than patients with lower SOST expressing tumors (Fig. 1B). In addition, IHC analysis of 69 pairs of primary breast cancer foci and corresponding metastatic lesions in multiple organs revealed that SOST expression increased in both primary foci and bone metastasis tissues, compared to normal breast tissue and breast cancer tissue without bone metastasis, while SOST expression was lower in the liver and lung metastatic lesions (Fig. 1C-D), supporting the relevance of SOST to breast cancer bone metastasis. Moreover, SOST expression was positively associated with N stage and distant postoperative metastases, especially those to the bone (*P* < 0.01) rather than the lung, liver, or brain (Table 1). However, there was no significant association with T stage, estrogen receptor (ER), progesterone receptor (PR), HER2 status, Ki67 status, molecular type, or menopause in this population (Table 1). Univariate and multivariate analyses unveiled that high SOST expression and menopause status, N stage or ER and PR positivity, but not T stage, molecular type, HER2 and Ki67 status, were independent risk factors for worse distant metastasis-free survival (DMFS) in this population (*P* < 0.01, Table 2). Hence, SOST may be a specific marker for breast cancer bone metastasis.

**Table 1:** Relationship between SOST expression and clinical pathological features of patients with breast cancer

	Total (*n* = 422)	SOST expression		*P-*value
		Low (%)	High (%)	
T stage				0.133
T1	123 (29.15)	88 (71.54)	35 (28.46)	
T2	283 (67.06)	222 (78.45)	61 (21.55)	
T3	16 (3.79)	10 (62.50)	6 (37.50)	
N stage				**0.006**
N0-1	328 (77.73)	259 (78.96)	69 (21.04)	
N2–3	94 (22.27)	61 (64.89)	33 (35.11)	
ER status				0.636
ER negative	149 (35.31)	111 (74.50)	38 (25.50)	
ER positive	273 (64.69)	209 (76.56)	64 (23.44)	
PR status				0.734
PR negative	200 (47.39)	150 (5.00)	50 (25.00)	
PR positive	222 (52.61)	170 (76.58)	52 (23.42)	
HER2 status				0.165
HER2 negative	251 (59.48)	184 (73.31)	67 (26.69)	
HER2 positive	171 (40.52)	136 (79.53)	35 (20.47)	
Ki67 status				0.570
Ki67 < 20%	193 (45.73)	149 (77.20)	44 (22.80)	
Ki67 ≥ 20%	229 (54.27)	171 (74.67)	58 (25.33)	
Molecular type				0.395
HR + HER2-	188 (44.55)	140 (74.47)	48 (25.53)	
HR + HER2 +	95 (22.51)	77 (81.05)	18 (18.95)	
HR-HER2 +	76 (18.01)	59 (77.63)	17 (22.37)	
TNBC	63 (14.93)	44 (69.84)	19 (30.16)	
Distant metastasis^a^				** < 0.001**
No	272 (64.45)	227 (83.46)	45 (16.54)	
Yes	150 (35.55)	93 (62.00)	57 (38.00)	
Bone metastasis				** < 0.001**
No	313 (74.17)	254 (81.15)	59 (18.85)	
Yes	109 (25.83)	66 (60.55)	43 (39.45)	
Lung metastasis				0.282
No	374 (88.63)	287 (76.74)	87 (23.26)	
Yes	48 (11.37)	33 (68.75)	15 (31.25)	
Liver metastasis				0.155
No	397 (94.08)	304 (76.57)	93 (23.43)	
Yes	25 (5.92)	16 (64.00)	9 (36.00)	
Brain metastasis				0.686
No	414 (98.10)	313 (75.60)	101 (24.40)	
Yes	8 (1.90)	7 (87.50)	1 (12.50)	
Menopause status				0.300
Premenopausal	172 (40.76)	135 (78.49)	37 (21.51)	
Postmenopausal	250 (59.24)	185 (74.00)	65 (26.00)	

Data are n (%)

*HR* Hormone receptor, *ER* Estrogen receptor, *PR* Progesterone receptor, *HER2* Human epidermal growth factor receptor 2, *TNBC* Triple-negative breast cancer

^a^ Distant metastasis: Cases with distant metastasis by the deadline of follow-up

**Table 2:** Analysis of risk factors for distant metastasis-free survival (DMFS)

	Univariate analysis		Multivariate analysis	
	Hazard ratio (95% CI)	*P-*value	Hazard ratio (95% CI)	*P*-value
SOST × menopause status	2.59 (1.80–3.73)	** < 0.001**	2.12 (1.46–3.08)	** < 0.001**
T stage		0.660		0.917
T2 vs. T1	1.02 (0.71–1.46)	0.926	1.00 (0.68–1.47)	0.988
T3 vs. T1	1.44 (0.65–3.21)	0.374	1.19 (0.51–2.79)	0.696
N stage	4.38 (3.16–6.06)	** < 0.001**	4.15 (2.94–5.88)	** < 0.001**
ER × PR	0.60 (0.43–0.83)	**0.002**	0.54 (0.35–0.82)	**0.004**
HER2	0.99 (0.72–1.38)	0.969	0.66 (0.38–1.16)	0.150
Ki67	0.98 (0.71–1.34)	0.877	0.98 (0.69–1.39	0.895
Molecular type		0.910		0.331
HR + HER2 +				
vs. HR + HER2-	0.90 (0.59–1.38)	0.645	0.68 (0.43–1.08)	0.103
HR-HER2 +				
vs. HR + HER2-	1.09 (0.70–1.69)	0.706	0.66 (0.38–1.16)	0.150
TNBC				
vs. HR + HER2-	0.96 (0.60–1.56)	0.879	0.82 (0.45–1.48)	0.503

*DMFS* Distant metastasis-free survival, *HR* Hormone receptor, *CI* Confidence interval, *ER* Estrogen receptor, *PR* Progesterone receptor, *HER2* Human epidermal growth factor receptor 2, *TNBC* Triple-negative breast cancer. × represents joint analysis

### SOST knockdown inhibits tumor progression and bone metastasis

**Fig. 2:**
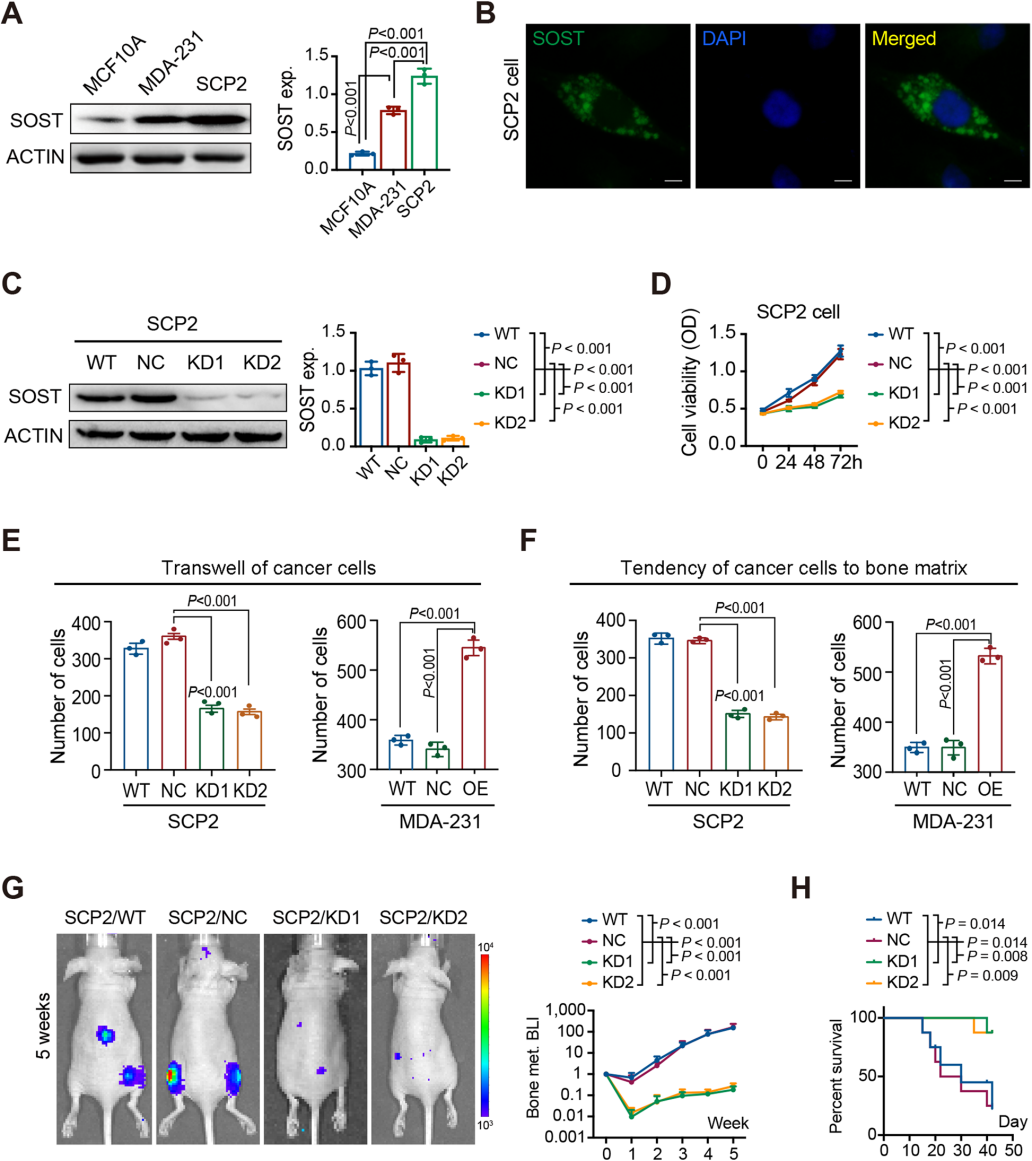
*SOST* knockdown inhibits the migration of breast cancer cells toward MC3T3-E1 cells and bone metastasis. **A** Western blot analysis of the expression of SOST in MCF10A, MDA-MB-231 and SCP2 cells. **B** Immunofluorescence analysis of SOST expression in SCP2 cells (SOST, green; DAPI, blue). **C** Western blot analysis of the efficiency of *shSOSTs* in SCP2 cells. **D** CCK-8 analysis of the effect of SOST silencing on the viability of SCP2 cells. **E** The migration of SOST-silencing SCP2 and SOST-over-expressing MDA-MB-231 cells toward MC3T3-E1 cells in vitro. **F** Adhesion of SOST-silencing SCP2 or SOST-over-expressing MDA-MB-231 cells onto the bone matrix. **G** Bioluminescent imaging of bone metastasis in BALB/c-nu mice at 5 weeks after intra-ventricular injection with wild-type (WT), negative control (NC) and *SOST* silencing SCP2 cells (*n* = 8–10 per group). **H** Survival of BALB/c-nu mice receiving WT, NC or KDs SCP2 cells (*n* = 8–10 per group)

Since primary tumors and corresponding metastatic tumor lesions share genetic similarities across multiple types of cancers, individual metastases probably arise from a single, dominant clone within the primary tumor [27]. Actually, breast cancer SCP2 cells were derived from a single MDA-MB-231 cell in mice after repeated left ventricular injection and had a potent propensity for bone metastasis [28]. To investigate the importance of SOST in the organotropism of cultured SCP2 cells, the levels of SOST expression in SCP2, MDA-MB-231 and MCF-7 cells were examined by Western blot (Fig. 2A). The relative levels of SOST expression in SCP2 cells were obviously higher than that in MDA-MB-231 and MCF-7 cells (Fig. 2A). Furthermore, immunofluorescent analysis revealed that SOST was mainly expressed in the cytoplasm of SCP2 cells (Fig. 2B).

To test the function of SOST, transduced with lentivirus for stable expression of shSOST significantly reduced SOST expression by 85% (*P* < 0.001, Fig. 2C). Functionally, SOST silencing significantly decreased the proliferation of SCP2 cells in vitro (Fig. 2D and S2A), but not their migration (Fig. S2B). Compared with SCP2/NC and unmanipulated SCP2 cells, SCP2/KD cells displayed significantly less capacity to migrate toward osteogenic precursor MC3T3-E1 cells while SOST over-expressing MDA-MB-231 cells exhibited stranger capacity to migrate toward MC3T3-E1 cells, relative to the control MDA-MB-231 cells (Fig. 2E and S2C). A similar pattern of cell adhesion onto the bone matrix layer was detected in different lines of breast cancer cells (Fig. 2F and S2D). In addition, left-ventricular cardiac injection with SCP2/KD cells dramatically decreased osteolytic lesions in mice (Fig. 2G) and prolonged their survival throughout the 5-week observation period compared with injection with SCP2/WT and SCP2/NC cells in vivo (Fig. 2H). Thus, SOST silencing inhibited the adhesion onto the bone matrix and bone metastasis in mice, suggesting that SOST may be a critical factor for breast cancer bone metastasis.

### SOST enhances STAT3 phosphorylation to promote the proliferation and chemotaxis of SCP2 cells by enhancing TGF-β and KRAS transcription

**Fig. 3:**
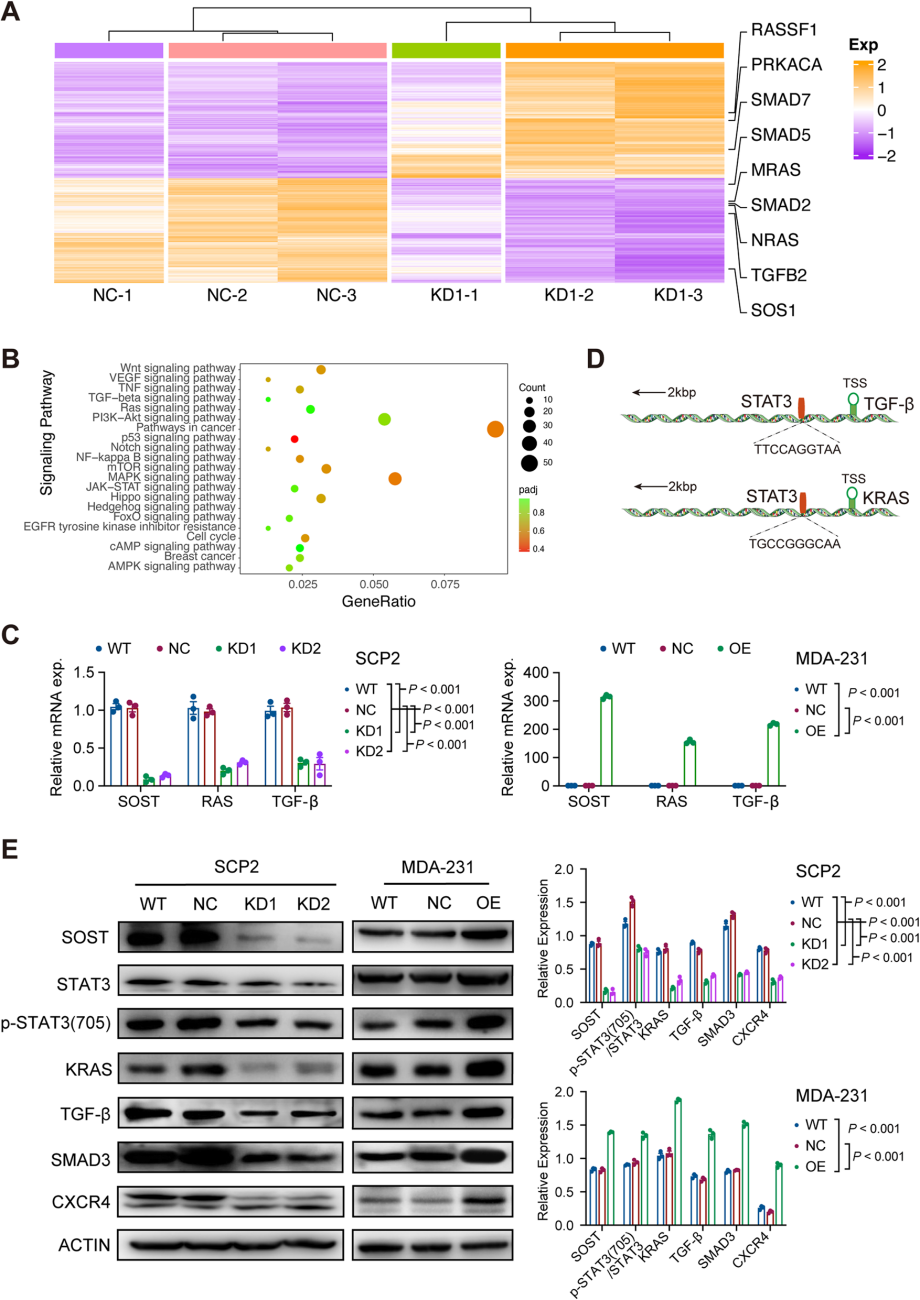
SOST promotes bone metastasis through the TGF-β/SMAD3 signaling. **A** Heatmap displayed the DEGs between the WT and SOST-silencing SCP2 cells after RNA-seq. **B** KEGG pathway enrichment of DEGs after *SOST* knockdown in SCP2 cells. **C** qRT-PCR analysis of the relative levels of *SOST*, *KRAS*, and *TGFB* mRNA transcripts in WT and SOST-silencing SCP2 cells or WT and SOST-over-expressing MDA-MB-231 cells. **D** A schematic diagram of the potential STAT3 binding sites in the *TGFB* and *KRAS* promoters predicted by rVista 2.0 software. **E** Western blot measurement of the relative levels of SOST, KRAS, TGF-β, SMAD3, CXCR4, and STAT3 to β-actin expression, and STAT3 phosphorylation in the indicated SCP2 cells or MDA-MB-231 cells

To explore the molecular mechanisms by which SOST affects the proliferation of SCP2 cells, the transcriptomes of SOST/NC and SOST/KD1 cells were analyzed by RNAseq and the DEGs in SOST/KD1 cells were analyzed by Cluster3.0 using lgRPKM values (Fig. 3A). KEGG enrichment was measured by gene ratio, FDR, and gene number (Fig. 3B). Apparently, SOST regulated the TGF-β/SMAD3 signaling and SOST silencing mitigated the relative levels of RAS and TGF-β mRNA transcripts in SCP2 cells (Fig. 3C). Considering the effect of SOST on breast cancer cell proliferation and the presence of SOST in the cytoplasm, we speculate that SOST may activate the RAS and TGF-β signaling pathways by interacting with intracytoplasmic transcription factors, such as STAT3. Next, the binding sites of STAT3 in the *KRAS* or *TGF-β* promotor regions were predicted using rVista 2.0 software according to the JASPAR [26] and are shown in Fig. 3D. In addition, analysis of STAT3-related genes using the GEPIA database revealed that the levels of *STAT3* expression were correlated positively with that of *KRAS*, *NRAS,* and *MRAS* (Fig. S3A), as well as with *TGFB2*, *SMAD2,* and *SMAD3* (Fig. S3B) in breast cancers.

Because the TGF-β/SMAD3 signaling can upregulate CXCR4 expression in breast cancer, promoting metastasis, and its ligand CXCL12 is enriched in the bone marrow microenvironment, we evaluated whether SOST silencing could change in the expression of these factors in SCP2 cells. We found that *SOST* silencing decreased the phosphorylation of STAT3 at Y705 and the levels of TGF-β, RAS, SMAD3 and CXCR4 expression in SCP2 cells (Fig. 3E). In contrast, induction of SOST over-expression led to opposite effects in low SOST-expressing MDA-MB-231 cells (Fig. 3E). Together, these results suggest that SOST in breast cancer cells may promote the proliferation of SCP2 cells by activating the TGF-β/RAS signaling, and promotes bone metastasis by upregulating CXCR4 expression.

### Discovery of small-molecule compounds that block the SOST pocket to disrupt the interaction of SOST with STAT3

**Fig. 4:**
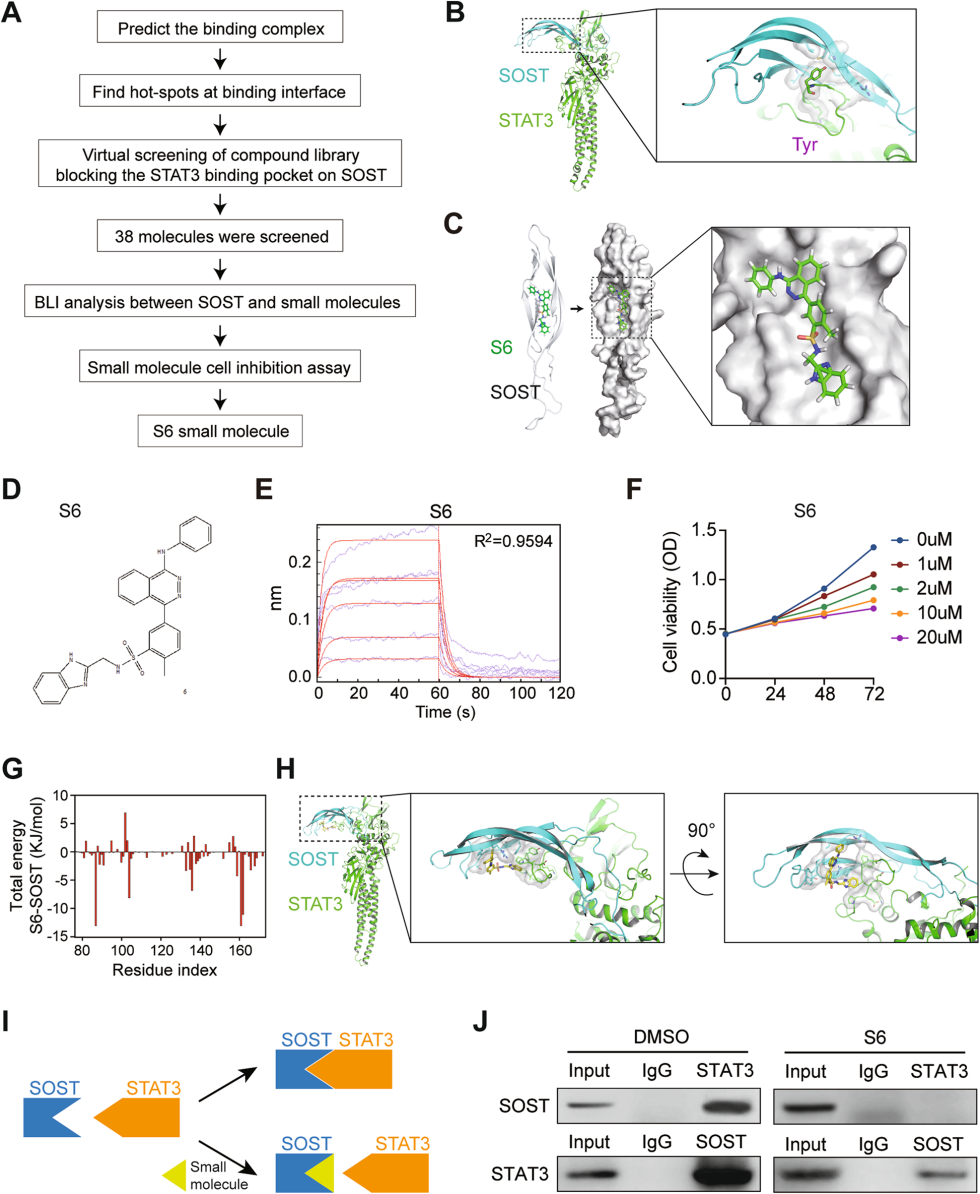
Screening and identification of S6, a small-molecule targeting SOST. **A** A scheme for computational screening of inhibitors that block STAT3 binding to SOST. **B** Protein–protein interface of SOST and STAT3. **C** Docking of S6 with the SOST pocket. **D** Structure of compound 6, named S6. **E** Multi-concentration gradient detection of S6 (KD = 3.992E-04, R^2^ = 0.9594). **F** Inhibitory effect of S6 on the viability of SCP2 cells. **G** Total energy of SOST-S6 complex. **H** Overall structure and close-up views of the SOST-S6-STAT3 complex. **I** A scheme for S6 blocking SOST-STAT3 binding. **J** Co-immunoprecipitation reveals that S6 inhibits the SOST-STAT3 binding in SCP2 cells

We next sought to model the interaction of SOST with STAT3 and identify its inhibitors from a small-molecule chemical library (Fig. 4A). First, our modeling suggested that a tyrosine residue (Y705) of STAT3 could dock in the pocket of SOST (Fig. 4B). Subsequently, we performed virtual screening for compounds that might block the binding of SOST to STAT3 (Fig. 4C). We screened approximately 120,000 compounds and obtained 38 candidates. We tested single-concentration of them in BLI test. Data from 35 of these compounds are shown in Figure S4 and SI Table 1, except for compounds 2, 10, and 25, which could not be detected due to poor solubility. Nine compounds (6, 11, 12, 17, 22, 24, 34, 38) were screened by multi-concentration gradients to obtain R2, *K*_d_, kdis, and response parameters (Fig. 4D-E, Fig. S5 and SI Table 2). Four compounds, 6, 11, 28, and 34, were selected for drug sensitivity testing (Fig. 4F and Fig. S5). It was notable that treatment with the compound 6, named as S6, significantly inhibited the proliferation of SCP2 cells in a dose and time-dependent manner (Fig. 4F). S6 boudn to SOST with a high affinity (Fig. 4G). Structural simulations indicated that S6 was deeply embedded in the SOST-STAT3 complex (Fig. 4H), and likely inhibited their interaction by blocking a pocket on SOST (Fig. 4I). Importantly, co-immunoprecipitation unveiled that treatment with S6 prevented the anti-STAT3-precipitated SOST and dramatically reduced the anti-SOST-precipitated STAT3 in SCP2 cells (Fig. 4J).

### S6 inhibits breast cancer progression

**Fig. 5:**
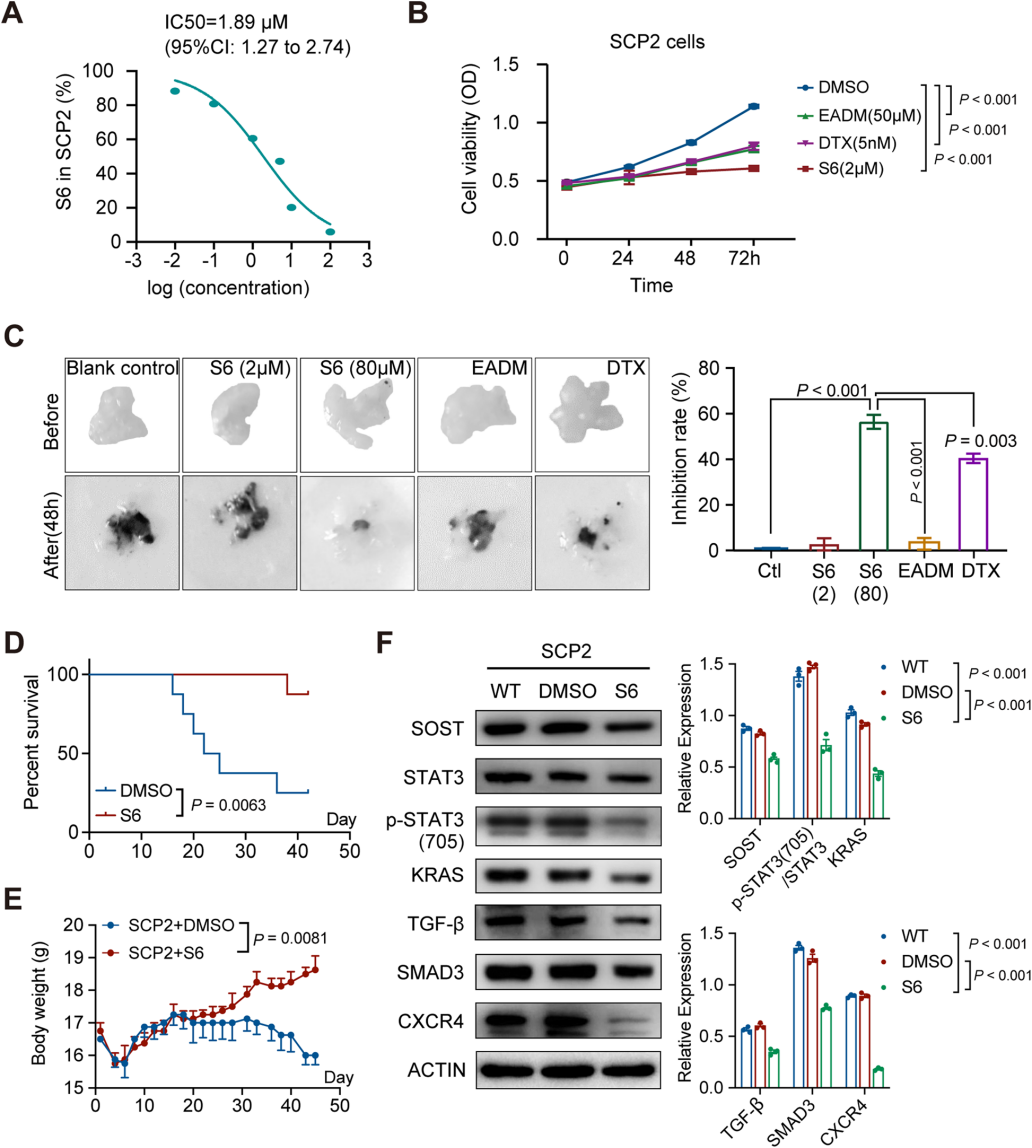
S6 inhibits the growth of breast cancer cells and tumors. **A** CCK8 assay analysis of half-maximal inhibitory concentration (IC_50_) of S6 for SCP2 cells (1.89 µM, 95%CI 1.27–2.74). **B** The effect of S6 (2 µM), DMSO, EADM (50 µM), and DTX (5 nM) on the viability of SCP2 cells. **C** Inhibition of S6 on the growth of breast-cancer organoids and the inhibition rates of treatment with 80 μM S6 for 48 h (38.96%, 95%CI 37.01 to 40.89), compared to that of 2 μM S6 for 48 h (0.53%, 95%CI 0.12 to 0.92), as well as EADM (500 µM) and DTX (250 nM). **D** Survival of SCP2 tumor-bearing mice after treatment with S6 or DMSO (*n* = 8–10 per group, *P* = 0.0063). **E** Body weights of SCP2 tumor-bearing mice after treatment with DMSO or S6 (10 mg/kg) for 45 days (*P* = 0.0081). **F** Western blot analysis of the relative levels of SOST, KRAS, TGF-β, SMAD3, CXCR4, and STAT3 expression and STAT3 phosphorylation in SCP2 cells after treatment with DMSO or S6 (2 µM) for 48 h

We further tested the anti-breast cancer effects in vitro and in vivo. S6 treatment significantly inhibited the proliferation of SCP2 cells with an IC_50_ of 1.89 µM (95%CI, 1.27 to 2.74) (Fig. 5A). The inhibitory effects of 2 μM S6 on breast cancer proliferation were superior to that of 50 μM EADM and 5 nM DTX in SCP2 cells (Fig. 5B), as well as in MCF-7 and MDA-MB-231 cells (Fig. S6A-B). Furthermore, treatment with S6 at 80 μM for 48 h inhibited the growth of breast-cancer organoids and its therapeutic effect was comparable to the conventional chemotherapy drugs of DTX or EADM (Fig. 5C). Finally, we tested whether treatment with S6 could modulate the bone metastasis of breast cancer in mice. Compared with control mice, treatment with S6 significantly increased the survival rate of tumor-bearing mice (Fig. 5D). Moreover, S6 treatment significantly increased body weight gain in mice (Fig. 5E). More importantly, there was no structural damage in vital organs of mice, suggesting that S6 treatment may be relatively safe in mice (Fig. S7). Treatment with S6 significantly decreased STAT3 phosphorylation and TGF-β, KRAS, SMAD3 and CXCR4 expression in SCP2 cells (Fig. 5F). Collectively, treatment with S6 inhibited STAT3 phosphorylation and attenuated the downstream TGF-β/KRAS signaling pathways, which in turn inhibited breast cancer progression.

### S6 inhibits breast cancer bone metastasis

**Fig. 6:**
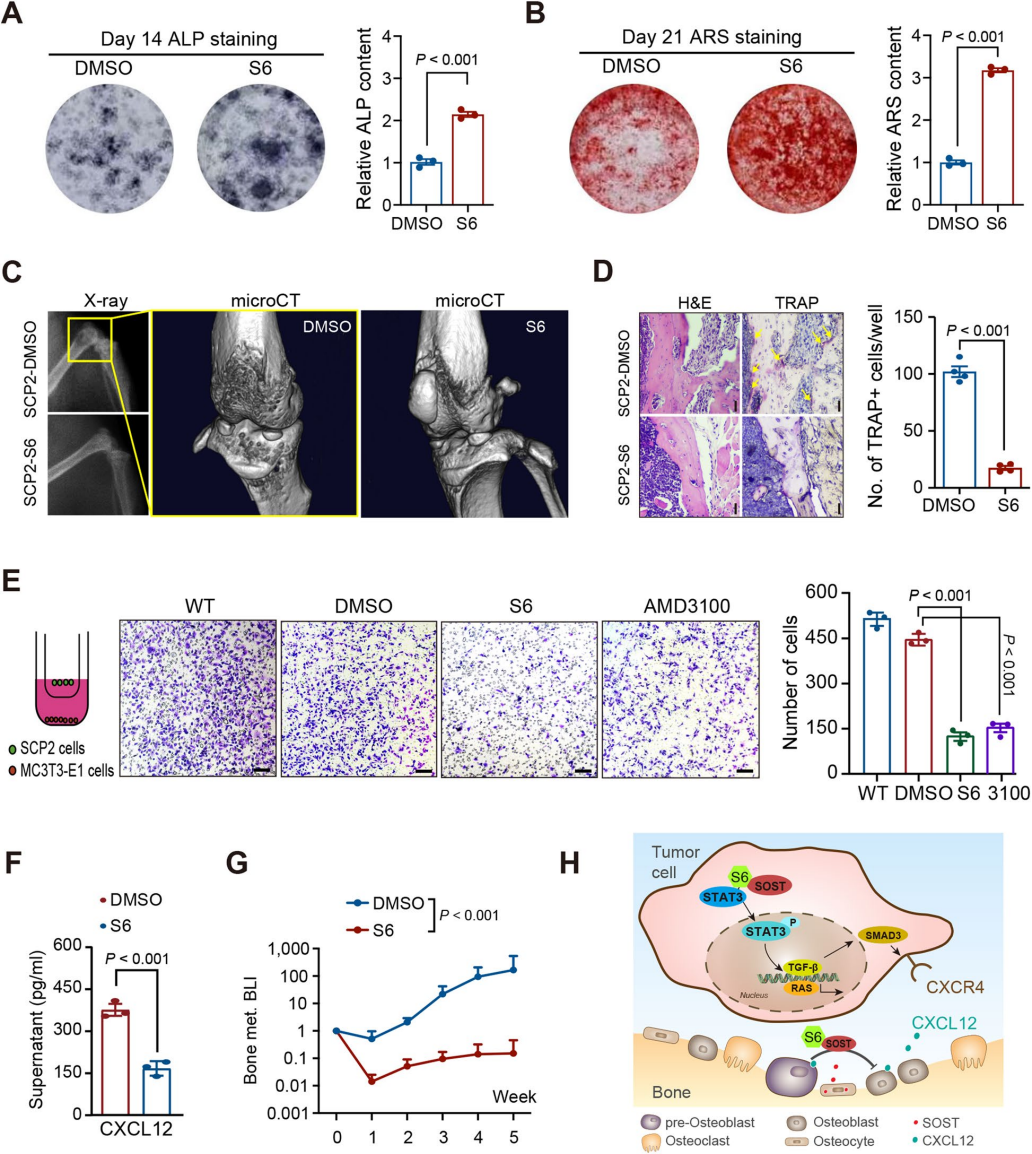
S6 inhibits bone metastasis of breast cancer cells and tumors. **A** ALP staining of osteoblast differentiation after S6 (2 µM) treatment for 14 days. **B** ARS staining of osteoblast differentiation after S6 (2 µM) treatment for 21 days. **C** Typical X-ray and microCT images of bone metastases in nude mice inoculated with SCP2 and treated with DMSO or S6 (10 mg/kg). **D** H&E and TRAP staining images of bone metastases in nude mice inoculated with SCP2 and treated with DMSO or S6. **E** Transwell migration assays revealed that treatment with S6 or AMD3100 (a CXCR4 inhibitor) inhibited SCP2 cell migration towards MC3T3-E1 cells. **F** treatment with S6 reduced the levels of CXCL12 in the supernatants of co-cultured SCP2 and MC3T3-E1 cells. **G** Bioluminescence imaging analysis of bone metastases in nude mice 5 weeks after intraventricular injection with SCP2 cells and treatment with DMSO or S6 (*n* = 8–10 per group). **H** A schematic diagram reveals that SOST binding to STAT3 activates the RAS and TGF-β/SMAD/CXCR4 signaling to promote cancer cell proliferation and osteotrophy, while S6 inhibits the SOST-STAT3 binding to inhibit the subsequent process

Finally, we tested the inhibitory effect of S6 on breast cancer bone metastasis. ALP and ARS staining exhibited that S6 treatment enhanced the differentiation of BMSCs (Fig. 6A and B). Furthermore, S6 treatment significantly inhibited the destruction of bone cortex in the tumor-bearing mice (Fig. 6C). H&E and TRAP staining revealed that S6 treatment reduced osteoclast activity in the tumor-bearing mice (Fig. 6D). Furthermore, administration of S6 or a CXCR4 inhibitor limited the migration of SCP2 cells toward MC3T3-E1 cells in vitro (Fig. 6E), and treatment with S6 also decreased CXCL12 concentrations in the supernatant of SCP2 co-cultured with MC3T3-E1 cells (Fig. 6F). In addition, S6 treatment reduced the rates of bone metastasis in the SCP2 tumor-bearing mice (Fig. 6G). Therefore, S6 blocks the STAT3 binding pocket of SOST, inhibiting the downstream signaling that regulates the proliferation and metastasis of SCP2 cells (Fig. 6H).

## Discussion

SREs are a major cause of morbidity and mortality in patients with cancers [29]. Therapy for bone metastasis includes external radiation therapy, chemotherapy, endocrine agents, targeted therapies, and radionucleotide-targeted therapy (NCCN 2022 version). While chemotherapy, bone-targeted agents (BTAs), and endocrine therapy have direct antitumor effects, bone-targeted agents, such as bisphosphonates and denosumab, act by blocking the response of host cells, mainly osteoclasts, to tumor products [29]. Regardless of the tissue of origin, osteoclasts are a key therapeutic target for treatment of patients with skeletal metastases [30]. BTAs for oncology include bisphosphonates, the RANKL inhibitor denosumab, and bone-seeking radionuclides including radium, strontium, and samarium. To date, these drugs primarily inhibit bone resorption, with no direct anti-cancer effect.

Identification of the phenotypic features of breast cancer bone metastases is an important step for understanding how to manage them. Despite the progressive increase in understanding this biological cascade, additional targeted agents are still needed to expand treatment options. Previous studies have shown elevated levels of circulating SOST in breast cancer patients, particularly in TNBC patients [14, 31]. In this study, we found that up-regulated SOST expression in breast cancer tissues was significantly associated with the development of breast cancer bone metastasis in a population of 422 breast cancer patients. This novel finding supports the notion that SOST acts as oncogenic factor to promote breast cancer bone metastasis [13, 14] and suggests that SOST may be a therapeutic target for inhibiting breast cancer bone metastasis.

For translation to clinical applications, SOST-neutralizing antibodies suppressed the bone metastatic microenvironment and prolonged the survival of tumor-bearing mice [13, 14]. However, there was no significant effect of anti-SOST on tumor growth. We selected breast cancer SCP2 cells that are prone to bone metastasis [32] to explore the molecular mechanisms and found that SOST promoted the proliferation of breast cancer cells by enhancing the STAT3/TGF-β/KRAS signaling. Our data provide a proof of concept that SOST is a promising target for the treatment of primary breast cancer foci and bone metastases.

Finally, we identified a compound that achieved robust therapeutic effects by disrupting the SOST-STAT3 interaction. The protein–protein interfaces (PPIs) are essential for biological processes, including tumorigenesis and cancer development [33]. Despite their importance in disease development, targeting PPIs was initially thought to be impossible due to their large, flat, and featureless interaction surfaces [34]. However, with recent technological breakthroughs, high-resolution structural studies have shown that not all residues at the PPIs are critical, but instead, a few “hot spots” confer most of the binding energy, paving the way for the development of inhibitors for PPIs [35, 36]. Consistent with this concept, our previous structural biology studies have shown that the SOST-STAT3 interaction is critically dependent on several key residues in SOST and STAT3, and that this interaction is potentially disrupted by small molecules.

Functionally, the compound S6 had tumor suppressive effects. Notably, the SOST monoclonal antibodies currently used for the treatment of osteoporosis primarily target loop 2 of SOST, which is involved in cardiovascular protection [37]. If a SOST inhibitor targets the loop 2 of SOST it will be associated with increased risk for the development of cardiovascular diseases, similar to romosozumab. In this study, we screened small-molecule compounds that potentially targeted a non-loop 2 pocket of SOST. As a result, S6 was very well tolerated in vivo with minimal toxicity, particularly avoiding cardiotoxicity. Structural modeling and immunoprecipitation of the S6-SOST complex confirmed the binding of S6 to SOST and its competition with STAT3 to disrupt the formation of SOST-STAT3 complex. At a molecular level, S6 appeared to occupy an important pocket of SOST to block its interaction with STAT3 in breast cancer cells.

In conclusion, our findings highlight the feasibility and therapeutic potential of targeting the SOST-STAT3 complex for the treatment of breast cancer bone metastases and identify S6 as a promising candidate for further development as a new class of cancer therapeutic agents.

## Supplementary Information

**Additional file 1:** **Fig. S1.** Survival analysis of SOST expression in different subgroups of breast cancer patients. **Fig. S2.** SOST knockdown inhibits the proliferation and migration of SCP2 cells in vitro. **Fig. S3.** Correlation analysis of STAT3 expression with RAS and TGF-β/SMADs. **Fig. S4.** BLI analysis of 35 small-molecule compounds targeting SOST, except for 3 insoluble small-molecule compounds. **Fig. S5.** Other candidates that targeting SOST protein. **Fig. S6.** The cytotoxicity of S6 compound against breast cancer cells. **Fig. S7.** The safety profile of S6 in the vital organs of tumor-bearing mice. **Table S1.** Single concentration screening of small-molecules. **Table S2.** The results of the multi-concentration gradient assays were calculated.  

## Data Availability

The datasets used and analysed during the current study are available from the corresponding author on reasonable request.

## Abbreviations

ALP: Alkaline phosphatase
ARS: Alizarin red staining
DEGs: Differentially expressed genes
BTAs: Bone targeted agents
BMSCs: Bone marrow mesenchymal stem cells
BSA: Bovine serum albumin
ELISA: Enzyme-linked immunosorbent assay
SOST: Sclerostin
STAT3: Signal transducer and activator of transcription 3
SREs: Skeletal-related events
TNBC: Triple-negative breast cancer
RANKL: Receptor activator of nuclear factor kappa-B ligand
IHC: Immunohistochemistry
HRP: Horseradish peroxidase
DMEM: Dulbecco’s modified Eagle’s medium
qRT-PCR: Quantitative real-time reverse transcription-polymerase chain reaction
MD: Molecular dynamics
RIPA: Radioimmunoprecipitation assay
SDS-PAGE: Sodium dodecyl sulfate polyacrylamide gel electrophoresis
TCGA: The Cancer Genome Atlas
