# Supplementary material for: Targeting SOST using a small-molecule compound retards breast cancer bone metastasis
Source: Mol Cancer. 2022 Dec 29;21:228. doi: 10.1186/s12943-022-01697-4 (PMC9798707; doi:10.1186/s12943-022-01697-4)
Supplement: Supplementary file 1 — Additional file 1: Fig. S1. Survival analysis of SOST expression in different subgroups of breast cancer patients. Fig. S2. SOST knockdown inhibits the proliferation and migration of SCP2 cells in vitro. Fig. S3. Correlation analysis of STAT3 expression with RAS and TGF-β/SMADs. Fig. S4. BLI analysis of 35 small-molecule compounds targeting SOST, except for 3 insoluble small-molecule compounds. Fig. S5. Other candidates that targeting SOST protein. Fig. S6. The cytotoxicity of S6 compound against breast cancer cells. Fig. S7. The safety profile of S6 in the vital organs of tumor-bearing mice. Table S1. Single concentration screening of small-molecules. Table S2. The results of the multi-concentration gradient assays were calculated. [file 12943_2022_1697_MOESM1_ESM.docx]

**Supplementary Materials**

**
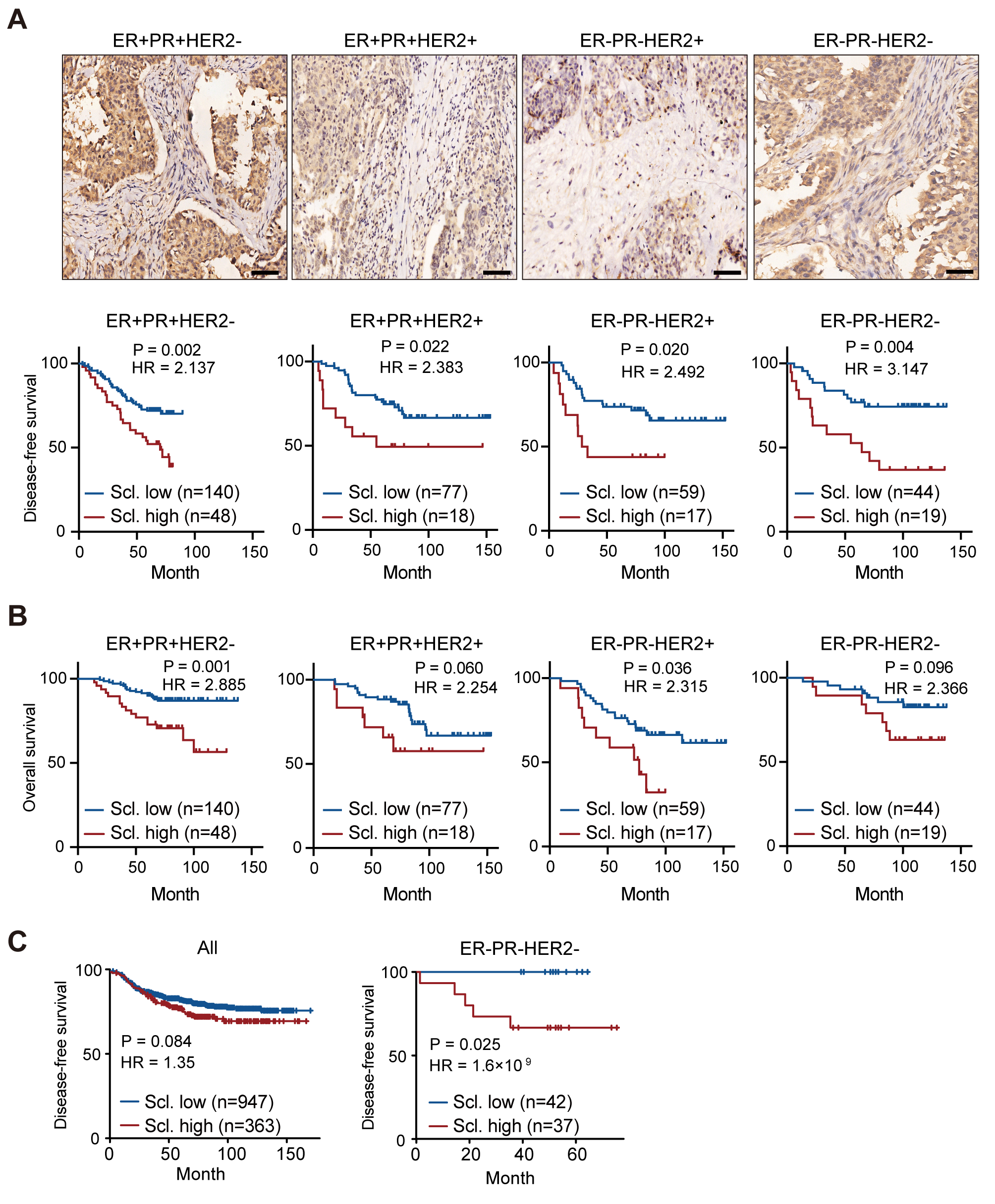
**

**Fig. S1. Survival analysis of SOST expression in different subgroups of breast cancer patients.**

(A and B) Subgroup survival analysis of disease-free survival (A) and overall survival (B) in four breast cancer subtypes with low versus high SOST expression based on data from our institution; scale bar, 100 µm.

(C) Disease-free survival of all 1,310 patients and 79 TNBC from TCGA database with low versus high SOST expression.


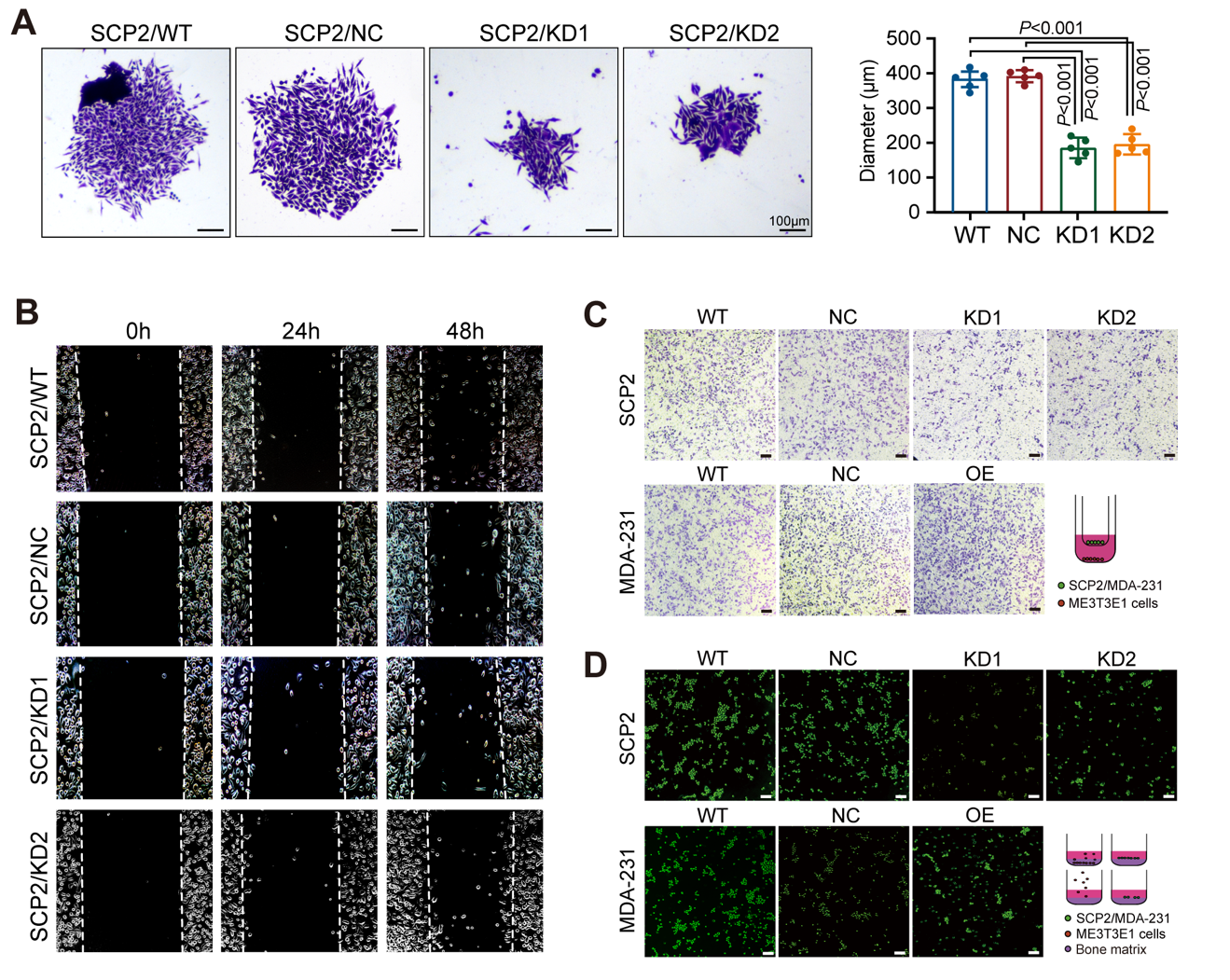


**Fig. S2. *SOST* knockdown inhibits the proliferation and migration of SCP2 cells *in vitro*.**

(A) Clone formation assay to detect cell proliferation after *SOST* knockdown in SCP2 cells; scale bar, 100 µm.

(B) Wound healing after *SOST* knockdown in SCP2 cells at 0, 24, and 48 h.

(C) Chemotaxis of SCP2 cells to MC3T3-E1 cells from SCP2 cells with *SOST* knockdown or MDA-MB-231 cells with *SOST* overexpression; scale bar, 100 µm.

(D) Adhesion to bone matrix from SCP2 cells with *SOST* knockdown or MDA-MB-231 cells with *SOST* overexpression; scale bar, 100 µm.


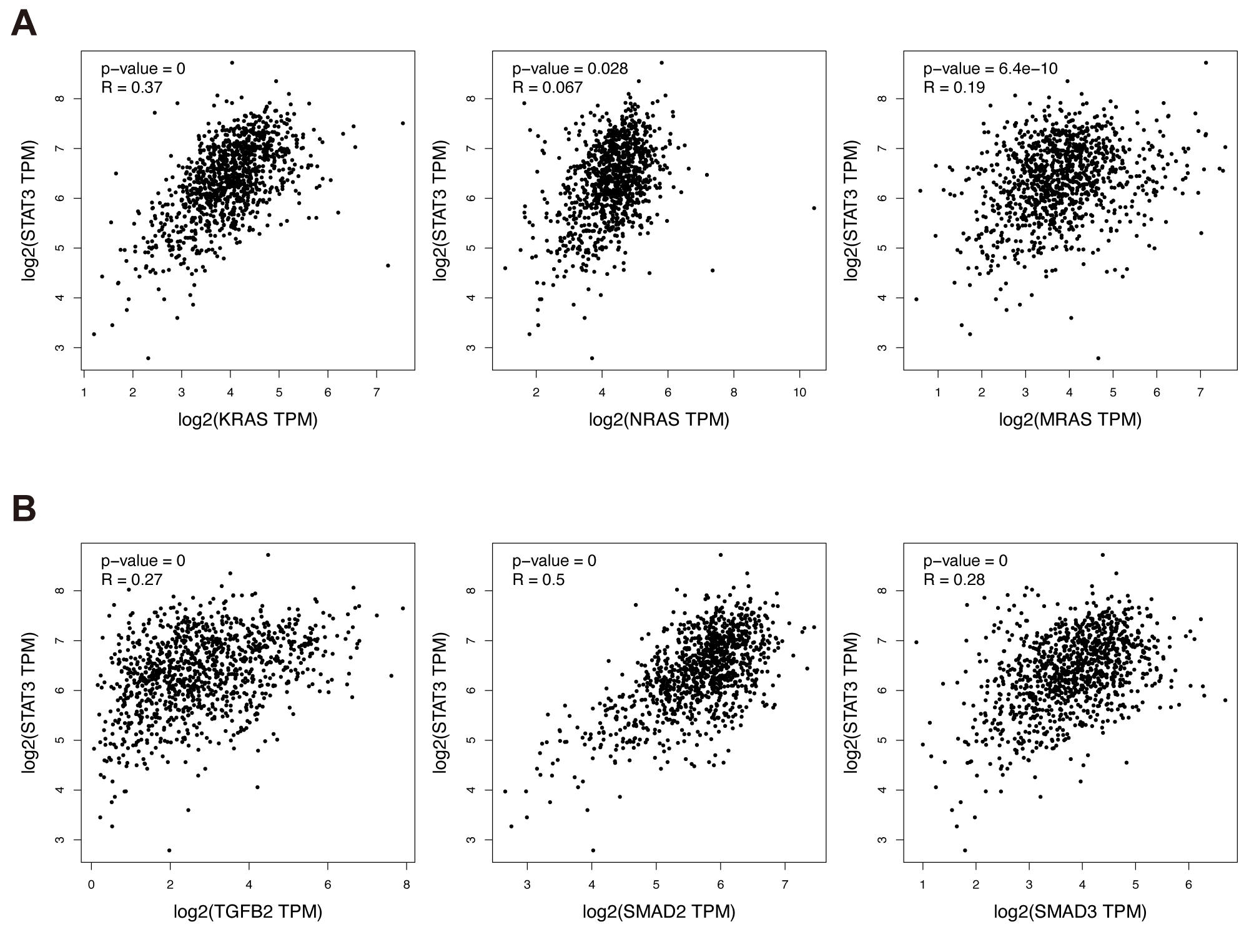


**Fig. S3. Correlation analysis of STAT3 expression with RAS and TGF-β/SMADs.**

1. Correlation of STAT3 with KRAS, NRAD and MRAS using GEPIA.
2. Correlation of STAT3 with TGFB2, SMAD2 and SMAD3 using GEPIA.

**
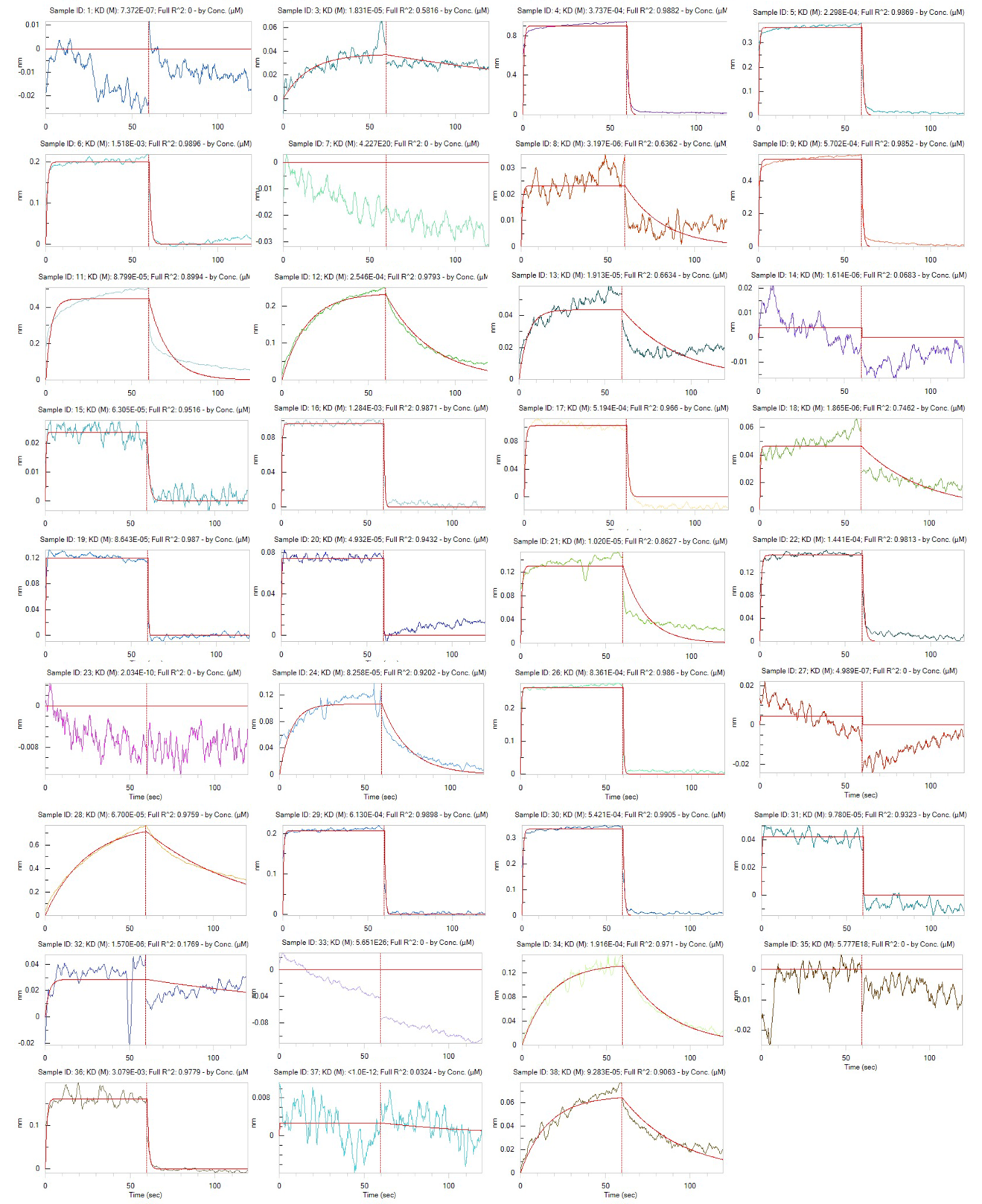
**

**Fig. S4. BLI analysis of 35 small-molecule compounds targeting SOST, except for 3 insoluble small-molecule compounds.**


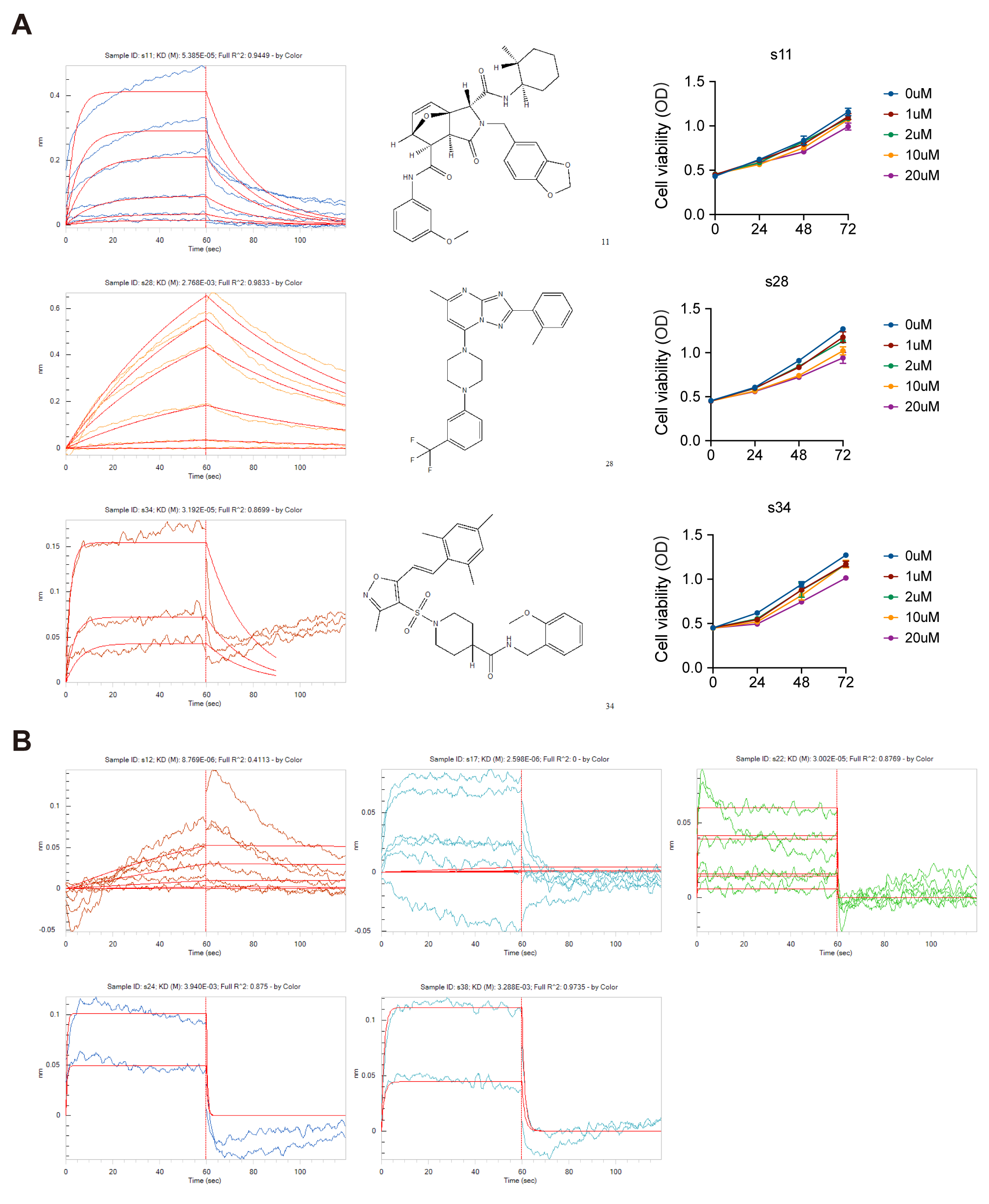


**Fig. S5. Other candidates that targeting SOST protein.**

1. Negative inhibition by S11, S28 and S34.
2. Multi-concentration gradient assays of S12, S17, S22, S24 and S38.

**
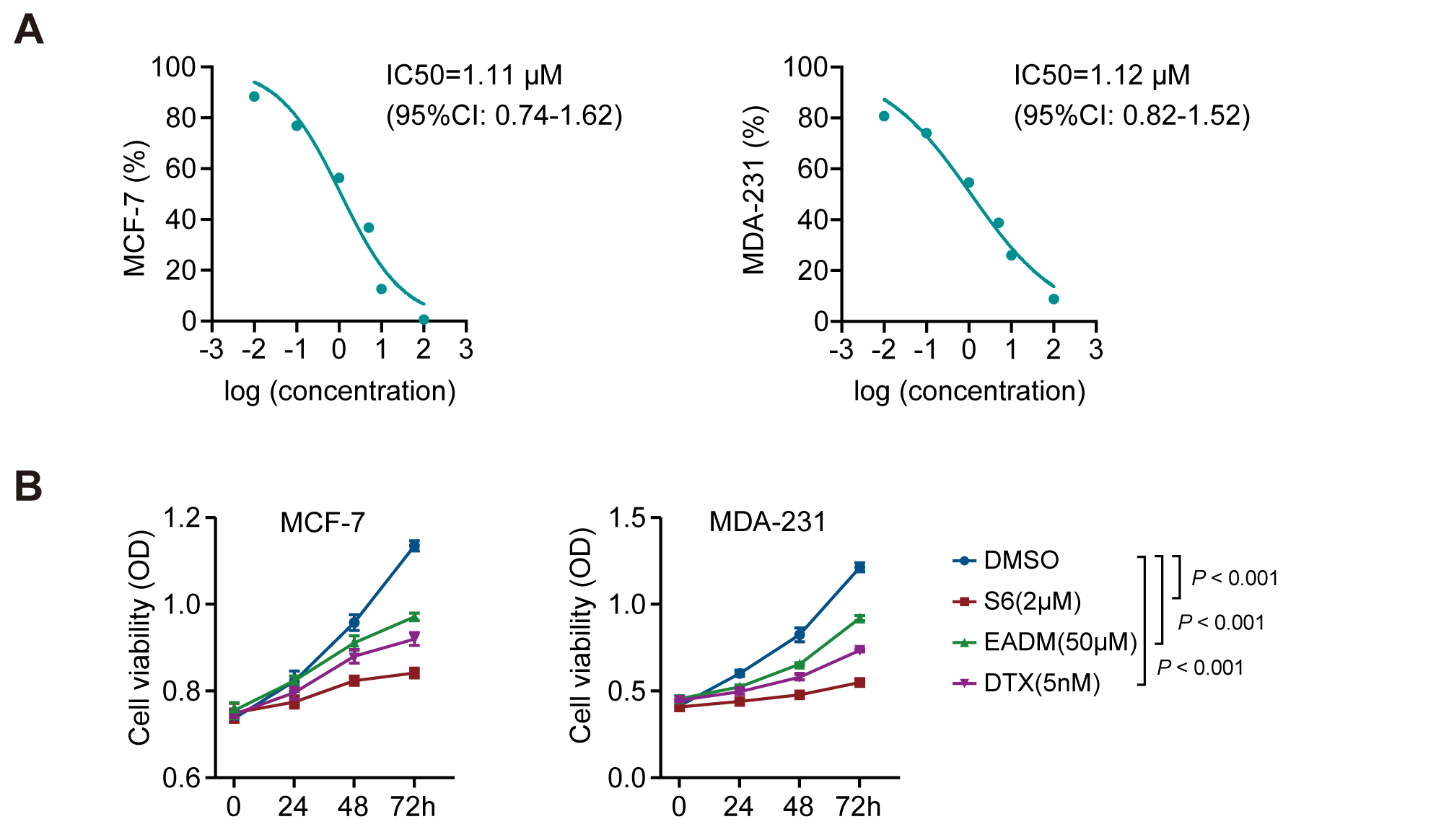
**

**Fig. S6. The cytotoxicity of S6 compound against breast cancer cells.**

(A) IC50 of S6 for MCF-7 (1.11 µM, 95%CI (0.74-1.62)) and MDA-MB-231 cell lines (1.12 µM, 95%CI (0.82-1.52)) by CCK8 assay.

(B) Inhibition of S6 (2 µM), as well as DMSO, EADM (50 µM) and DTX (5 nM) on MCF-7 and MDA-MB-231 viability (P<0.001).


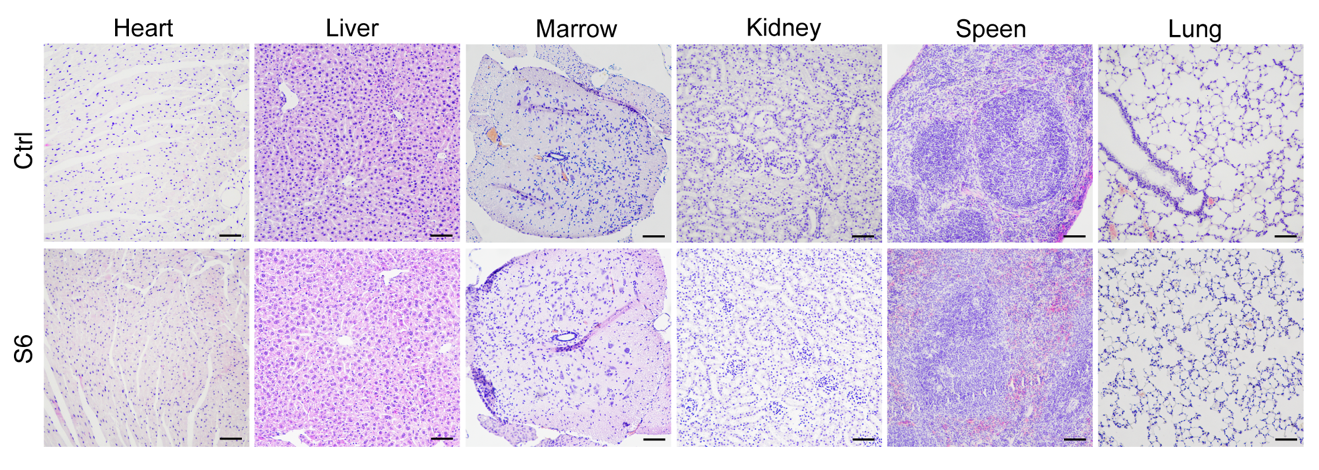


**Fig. S7. The safety profile of S6 in the vital organs of tumor-bearing mice.**

H&E staining of organ sections from nude mice treated with or without S6, including heart, liver, marrow, kidney, spleen, and lung (n=8-10 per group; scale bar, 100 µm).

**Table S1. Single concentration screening of small-molecules.**

Nine compounds (6, 11, 12, 17, 22, 24, 34, 38) were screened for multi- concentration gradient validation by information on R^2^, *K*_d_, kdis and Response parameters.

| Sample ID | Conc. (µM) | Response | KD (M) | kon(1/Ms) | kdis(1/s) | Full R^2 |
| --- | --- | --- | --- | --- | --- | --- |
| 1 | 148.8 | -0.0216 | 7.37E-07 | 1.00E-06 | <1.0E-07 | 0 |
| 3 | 167 | 0.0381 | 1.83E-05 | 3.63E+02 | 6.65E-03 | 0.5816 |
| 4 | 160.8 | 0.9335 | 3.74E-04 | 2.96E+03 | 1.11E+00 | 0.9882 |
| 5 | 170.5 | 0.3723 | 2.30E-04 | 4.73E+03 | 1.09E+00 | 0.9869 |
| **6** | **125** | **0.2049** | **1.52E-03** | **6.44E+02** | **9.77E-01** | **0.9896** |
| 7 | 201.7 | -0.0203 | 4.23E+20 | 7.75E-01 | 3.27E+20 | 0 |
| 8 | 91.1 | 0.03 | 3.20E-06 | 1.44E+04 | 4.60E-02 | 0.6362 |
| 9 | 189.3 | 0.5534 | 5.70E-04 | 2.09E+03 | 1.19E+00 | 0.9852 |
| **11** | **231** | **0.4986** | **8.80E-05** | **1.02E+03** | **8.93E-02** | **0.8994** |
| **12** | **216.5** | **0.2398** | **2.55E-04** | **1.48E+02** | **3.76E-02** | **0.9793** |
| 13 | 103.8 | 0.0546 | 1.91E-05 | 1.58E+03 | 3.02E-02 | 0.6634 |
| 14 | 120 | -0.0006 | 1.61E-06 | 2.47E+94 | 3.98E+88 | 0.0683 |
| 15 | 131 | 0.0235 | 6.31E-05 | 1.20E+04 | 7.59E-01 | 0.9516 |
| 16 | 170.2 | 0.1002 | 1.28E-03 | 1.53E+03 | 1.97E+00 | 0.9871 |
| **17** | **148.7** | **0.1019** | **5.19E-04** | **1.91E+03** | **9.91E-01** | **0.966** |
| 18 | 172.6 | 0.0552 | 1.87E-06 | 1.45E+04 | 2.71E-02 | 0.7462 |
| 19 | 182.7 | 0.1156 | 8.64E-05 | 4.58E+04 | 3.95E+00 | 0.987 |
| 20 | 131 | 0.0752 | 4.93E-05 | 3.62E+05 | 1.79E+01 | 0.9432 |
| 21 | 161.5 | 0.1452 | 1.02E-05 | 7.33E+03 | 7.48E-02 | 0.8627 |
| **22** | **97** | **0.1502** | **1.44E-04** | **5.86E+03** | **8.45E-01** | **0.9813** |
| 23 | 120 | -0.0094 | 2.03E-10 | 2.48E-06 | <1.0E-07 | 0 |
| **24** | **107.7** | **0.119** | **8.26E-05** | **7.75E+02** | **6.40E-02** | **0.9202** |
| 26 | 137.2 | 0.2724 | 8.36E-04 | 2.65E+03 | 2.22E+00 | 0.986 |
| 27 | 46.4 | -0.0039 | 4.99E-07 | 3.32E+123 | 1.66E+117 | 0 |
| **28** | **97.6** | **0.7192** | **6.70E-05** | **2.48E+02** | **1.66E-02** | **0.9759** |
| 29 | 88.1 | 0.2123 | 6.13E-04 | 2.89E+03 | 1.77E+00 | 0.9898 |
| 30 | 138.8 | 0.3419 | 5.42E-04 | 2.35E+03 | 1.28E+00 | 0.9905 |
| 31 | 147.2 | 0.0396 | 9.78E-05 | 1.89E+05 | 1.85E+01 | 0.9323 |
| 32 | 94.9 | -0.001 | 1.42E-04 | 2.93E+05 | 4.15E+01 | 0 |
| 32 | 94.9 | 0.0223 | 1.57E-06 | 4.50E+03 | 7.07E-03 | 0.1769 |
| 33 | 109.8 | -0.0368 | 5.65E+26 | 2.71E+00 | 1.53E+27 | 0 |
| **34** | **123.2** | **0.1284** | **1.92E-04** | **1.95E+02** | **3.73E-02** | **0.971** |
| 35 | 190.7 | 0.0004 | 5.78E+18 | 4.00E-04 | 2.31E+15 | 0 |
| 36 | 115.6 | 0.1625 | 3.08E-03 | 3.39E+02 | 1.04E+00 | 0.9779 |
| 37 | 159.1 | -0.0029 | <1.0E-12 | 1.17E+123 | 1.52E-02 | 0.0324 |
| **38** | **106.5** | **0.0693** | **9.28E-05** | **3.13E+02** | **2.90E-02** | **0.9063** |

**Table S2. The results of the multi-concentration gradient assays were calculated.**

Four small-molecules, 6, 11, 28 and 34, were preferentially selected for the drug sensitivity test.

| Sample ID | KD (M) | kon(1/Ms) | kdis(1/s) | Full R^2 |
| --- | --- | --- | --- | --- |
| s6 | 3.99E-04 | 7.65E+02 | 3.05E-01 | 0.9594 |
| s11 | 5.39E-05 | 1.00E+03 | 5.40E-02 | 0.9449 |
| s12 | 8.77E-06 | 4.62E+01 | 4.05E-04 | 0.4113 |
| s17 | 2.60E-06 | 6.73E+01 | 1.75E-04 | 0 |
| s22 | 3.00E-05 | 7.37E+05 | 2.21E+01 | 0.8769 |
| s24 | 3.94E-03 | 4.79E+02 | 1.89E+00 | 0.875 |
| s28 | 2.77E-03 | 5.21E+00 | 1.44E-02 | 0.9833 |
| s34 | 3.19E-05 | 1.81E+03 | 5.78E-02 | 0.8699 |
| s38 | 3.29E-03 | 2.49E+02 | 8.18E-01 | 0.9735 |
